# Inhibition of *Cronobacter sakazakii* Virulence Factors by Citral

**DOI:** 10.1038/srep43243

**Published:** 2017-02-24

**Authors:** Chao Shi, Yi Sun, Zhiyuan Liu, Du Guo, Huihui Sun, Zheng Sun, Shan Chen, Wenting Zhang, Qiwu Wen, Xiaoli Peng, Xiaodong Xia

**Affiliations:** 1 College of Food Science and Engineering, Northwest A&F University, Yangling, Shaanxi 712100, China; 2 Sino-US Joint Research Center for Food Safety, Northwest A&F University, Yangling, Shaanxi 712100, China

## Abstract

*Cronobacter sakazakii* is a foodborne pathogen associated with fatal forms of necrotizing enterocolitis, meningitis and sepsis in neonates and infants. The aim of this study was to determine whether citral, a major component of lemongrass oil, could suppress putative virulence factors of *C. sakazakii* that contribute to infection. Sub-inhibitory concentrations of citral significantly decreased motility, quorum sensing, biofilm formation and endotoxin production. Citral substantially reduced the adhesion and invasion of *C. sakazakii* to Caco-2 cells and decreased bacterial survival and replication within the RAW 264.7 macrophage cells. Citral also repressed the expression of eighteen genes involved in the virulence. These findings suggest that citral has potential to be developed as an alternative or supplemental agent to mitigate the infections caused by *C. sakazakii*.

*Cronobacter* (formerly *Enterobacter sakazakii*) is a Gram-negative, motile, peritrichous, non-spore forming and rod-shaped opportunistic bacterial pathogen^1^ and consists of seven species^2^. Among these species, *C. sakazakii* is primarily associated with such neonatal infections as bacteremia, necrotizing enterocolitis (NEC) and infant meningitis. Fatality rates of 50–80% are reported for infected neonates and infants, and 20% of survivors develop serious neurological disorders^3^. Among older children and adults, NEC is characterized by the colonization of the gastrointestinal lumen with reported fatality rates of 10–55%^4^.

Consumption of powered infant formula (PIF) is considered to be the primary route for *C. sakazakii* in neonatal infections^5^. As an orally ingested pathogen that causes systemic infections, *C. sakazakii* must adhere to the host cell, cross the intestinal epithelial cells, enter into the blood stream and transcytose across the blood–brain barrier^6^. Therefore, the ability of *C. sakazakii* to adhere to and invade the intestinal epithelium is the first step for its pathogenesis. Previous studies demonstrated that *C. sakazakii* efficiently adhered to and invaded human epithelial cells, specifically Caco-2 *in vitro*^7^. Moreover, *C. sakazakii* was demonstrated to survive and replicate within macrophages^8^. *C. sakazakii* could tolerate the intracellular environment of macrophages and use macrophages as a vehicle to invade the other body organs.

Bacterial biofilms provide a physical barrier and protect cells against a variety of environmental stresses such as UV light, desiccation and treatment with antimicrobial and sanitizing agents^9^,^10^. Previous studies confirmed that *C. sakazakii* form biofilms on silicon, latex, stainless steel surfaces, preparation areas and clinical settings as well as neonatal nasogastric feeding tubes used in infant-feeding equipment^11^,^12^. Washing with water or sanitizers do not always eliminate *C. sakazakii* on abiotic surfaces, presumably because of its existence in biofilms^13^.

Quorum sensing (QS), used by bacterial pathogens to coordinate the expression of virulence factors at high cell densities, is a key regulator governing various physiological processes such as biofilm formation, bioluminescence, motility, and other virulence factor production^14^. Endotoxin (LPS) is an important component of the outer membrane of Gram-negative bacteria^15^. Upon ingestion, LPS may increase the permeability of the neonatal intestinal epithelium and induce potent pathophysiological effects in the host^6^.

With alarming increase of antibiotic resistance in various microbial pathogens, it is important to identify alternative strategies and novel agents to counter bacterial infections^16^. Currently, increasing attention have been focused on natural plant phytochemicals, which are thought less prone to induce bacterial resistance and are generally safe for human consumption^17^. Citral is a major active component of citrus oils, which are already commonly used in foods and beverages (e.g., soft drinks and deserts)^18^. Citral is approved for use in foods by the Food and Drug Administration (FDA, GRAS, 21 CFR 182·60). In addition, citral has been proven to exhibit antibacterial activity on *Escherichia coli, Salmonella* and *Listeria monocytogenes*^19^,^20^,^21^.

Although most of previous studies focused on the antimicrobial properties of citral, a limited number of studies examined its anti-virulence potential, even less information is available regarding its effect on reducing pathogenic potential of *C. sakazakii*. The purpose of this study is to investigate the effect of citral at sub-inhibitory concentrations (SICs) on the reduction of the virulence properties of *C. sakazakii*. Changes in motility, specific biofilm formations, QS, endotoxin production, adhesion and invasion of Caco-2 cells and survival and replication in macrophage cells were analyzed. In addition, expression of different virulence genes were determined by reverse-transcriptase (RT) PCR.

## Results

### Minimum Inhibitory Concentrations and Sub-Inhibitory Concentrations

**Figure 1:**
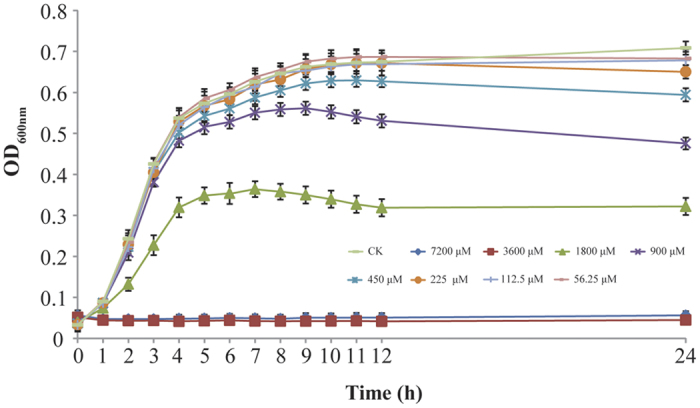
Growth of *C. sakazakii* ATCC 29544 in TSB with various concentrations of citral. Each value represents the average of three independent measurements.

Citral exhibited inhibitory effects against three tested *C. sakazakii* strains. Complete growth inhibition was achieved after 24 hours by citral at 3600 μM as measured by OD_600_ (Fig. 1). Thus the minimum inhibitory concentration (MIC) of citral against *C. sakazakii* strains was considered to be 3600 μM. Moreover, concentrations below 225 μM (1/16 MIC) exhibited no inhibition against *C. sakazakii* ATCC 29544 (Fig. 1) and those concentrations were further chosen as SICs to study the effects of citral on *C. sakazakii* virulence. After determining that citral was equally effective against three *C. sakazakii* isolates (ATCC 29544, ATCC 29004 and 7–17), we selected strain ATCC 29544 for further experiments.

### Motility

**Figure 2:**
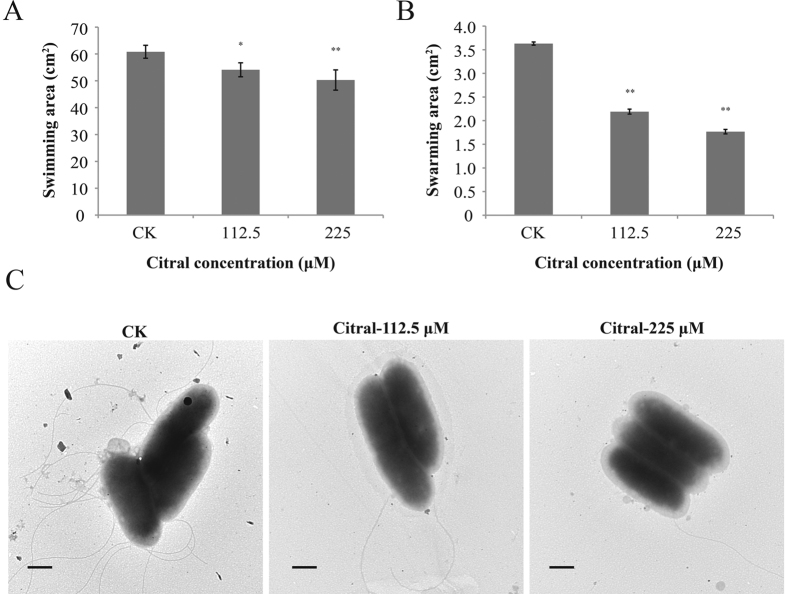
Effect of citral on motility of *C. sakazakii* ATCC 29544. (**A**) Effect of citral on swimming motility of *C. sakazakii* cells in 0.3% soft agar plates. (**B**) Effect of citral on swarming motility of *C. sakazakii* cells in 0.5% soft agar plates. Bars represent the standard deviation (n = 3). **P ≤ 0.01, *P ≤ 0.05. (**C**) Electron micrograph of *C. sakazakii* ATCC 29544 (bar, 500 nm). Bacterial cells were taken from swimming plates without citral, with 112.5 μM citral, and with 225 μM citral.

The effect of citral on *C. sakazakii* motility is shown in Fig. 2. Citral reduced both swimming and swarming ability of *C. sakazakii* ATCC 29544 (Fig. 2A,B). The original swimming area of *C. sakazakii* ATCC 29544 was 60.84 ± 2.41 cm^2^. Addition of citral at 225 μM and 112.5 μM caused swimming area reductions to 50.31 ± 3.77 cm^2^ (*P* ≤ 0.01) and 54.13 ± 2.61 cm^2^ (*P* ≤ 0.05) respectively. Swarming motility was also greatly impacted by citral. The original swarming area of *C. sakazakii* ATCC 29544 was 3.63 ± 0.03 cm^2^. Citral at 225 μM and 112.5 μM caused swarming area reductions to 1.77 ± 0.05 cm^2^ (*P* ≤ 0.01) and 2.19 ± 0.05 cm^2^ (*P* ≤ 0.01) respectively.

To examine the possible explanation for the effect of citral on motility of *C. sakazakii* ATCC 29544, we determined by electron transmission microscopy (TEM) whether there are changes in flagella after treatment with citral. The TEM images revealed that structurally intact flagellar filaments were observed on *C. sakazakii* ATCC 29544 cells obtained from the edge of swimming halos (Fig. 2C). *C. sakazakii* strains treated with citral exhibited less flagella than control cells (Fig. 2C).

### Biofilm Formation

The anti-biofilm efficacy of citral was investigated on *C. sakazakii* ATCC 29544. Bacteria were grown at 12 °C and 25 °C for 24, 48 and 72 hours on microtiter plates. Compared with the control, cells treated with citral showed a significant and dose-dependent inhibition of biofilm formation of *C. sakazakii*. The biofilm formation was inhibited by 67.1%, 69.5% and 70.1% with 225 μM citral after treatment at 25 °C for 24, 48 and 72 hours, respectively (Table 1).

### Quorum Sensing

**Figure 3:**
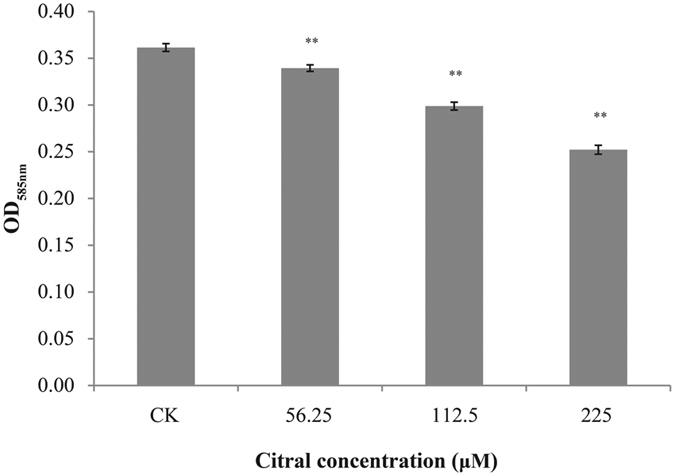
Inhibition of violacein production by *C. violaceum* ATCC 12472 at different concentrations of citral. Bars represent the standard deviation (n = 3).**P ≤ 0.01.

The effects of citral on *C. sakazakii* QS were indirectly assessed using *C. violaceum* 12472. This bacterium produces the pigment violacein in response to QS signal homologous to those produced by *C. sakazakii.* Citral showed no apparent antimicrobial activity against *C. violaceum* at concentrations used in this study (56.25 μM, 112.5 μM and 225 μM) with a broth micro-dilution method (data not shown). As seen in Fig. 3, anti-QS activity was shown when citral was used at 56.25 μM, 112.5 μM and 225 μM, as evidenced by a decreased production of violacein (about 94.44%, 83.33% and 69.44% of the control level, respectively).

### Adhesion and Invasion of Caco-2 cells

**Figure 4:**
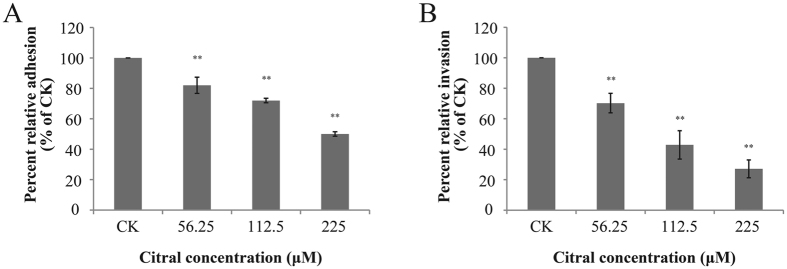
Effect of citral on adhesion (**A**) and invasion (**B**) of Caco-2 cells by *C. sakazakii* ATCC 29544. Bars represent the standard deviation (n = 3).**P ≤ 0.01.

The effect of citral on the adhesion and invasion of *C. sakazakii* ATCC 29544 in Caco-2 cells is depicted in Fig. 4. Citral at 56.25 μM, 112.5 μM and 225 μM inhibited adhesion (*P* ≤ 0.01) of *C. sakazakii* to 82%, 72% and 50% of the control, respectively (Fig. 4A). Citral was also effective in inhibiting (*P* ≤ 0.01) the ability of *C. sakazakii* to invade Caco-2 cells (Fig. 4B). The invasiveness of *C. sakazakii* ATCC 29544 was reduced by 27%, 43% and 70% with 56.25 μM, 112.5 μM and 225 μM of citral, respectively.

### Survival and Replication in RAW 264.7 cells

**Figure 5:**
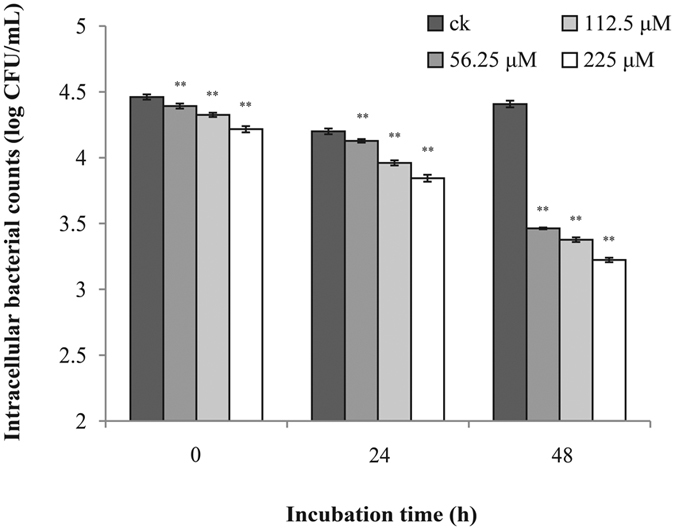
Effect of citral on bacterial intracellular survival and replication of *C. sakazakii* ATCC 29544 within macrophages. Bars represent the standard deviation (n = 3). **P ≤ 0.01.

Macrophages were able to kill *C. sakazakii* as demonstrated by the decrease cell population in citral-untreated samples (Fig. 5). The results showed all three concentrations (56.25 μM, 112.5 μM and 225 μM) of citral were able to significantly (*P* ≤ 0.01) decrease survival and replication of *C. sakazakii* in the macrophages in a dose-dependent manner during 48 hours (Fig. 5) when compared to the control medium.

### Endotoxin

**Figure 6:**
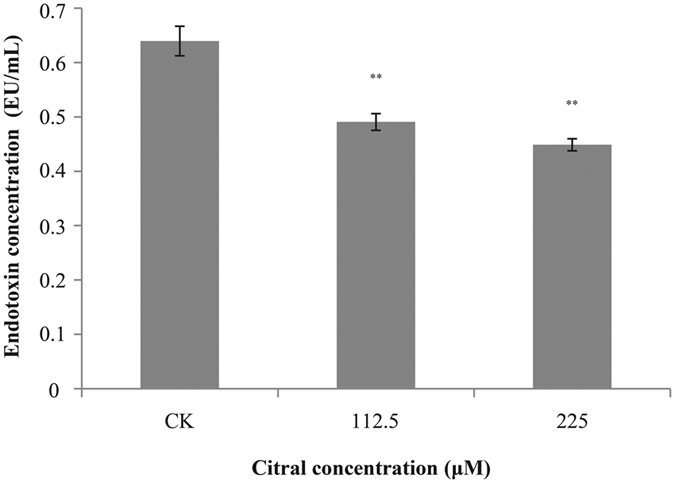
Effect of citral on endotoxin production by *C. sakazakii* ATCC 29544. Bars represent the standard deviation (n = 3). **P ≤ 0.01.

A good linearity was established between the absorbance intensity at 585 nm and the concentration of endotoxin (y = 0.377x + 0.021; R^2^ = 0.98). After the addition of citral to the cells, decreased endotoxin concentration was detected (Fig. 6). The original endotoxin concentration of *C. sakazakii* ATCC 29544 was 0.64 ± 0.03 EU/mL. Addition of citral at 112.5 μM and 225 μM caused a significant fall (*P* ≤ 0.01) in *C. sakazakii* endotoxin concentration to 0.49 ± 0.02 and 0.45 ± 0.01 EU/mL.

### Virulence-associated Genes

RT-qPCR results demonstrated that citral significantly decreased the expression of eighteen virulence-related genes in *C. sakazakii* (Table 2). Citral down-regulated the expression of *fliD, flhD*, and *flgJ* genes (critical for flagella regulation) to various degrees. Other down-regulated genes were *ompA* (outer membrane protein A), *ompX* (outer membrane protein X), *uvrY* (adherence and invasion), *motA, motB* (flagellar motor protein), *sod* (survival in macrophages), *bcsA* (cellulose synthase catalytic subunit), *bcsG* (cell biosynthesis and biofilm formation), *galE* (colanic acid synthesis), *kpsT* (K-antigen synthesis), *lpx, wzx* (LPS biosynthesis), and *luxR* (LuxR family transcriptional regulator). Citral also down-regulated the expression of two putative virulent plasmid associated genes: *iuc* (iron transport and regulation) and *eit* (iron uptake and siderophore system). Citral at 225 μM of citral was more effective than 112.5 μM in suppressing the expression of virulence genes (Table 2). Among the various virulence genes assayed, the expression of genes encoding *C. sakazakii* flagella, colanic acid, K-antigen and LPS were highly down-regulated in the presence of citral.

## Discussion

Bacterial motility is a complex process that employs mechanisms such as swimming and swarming that contribute to the subsequent formation of biofilm and is instrumental in host-microbial interactions and pathogenesis^22^. Both motility systems are directly mediated by a surface appendage, flagella, which play a role in the initial phase of biofilm development^23^. In the present study, it was demonstrated that citral greatly reduced two important types of motility in *C. sakazakii*. Additionally, RT-qPCR data showed that citral substantially decreased the expression of *C. sakazakii* genes associated with flagella structure and biosynthesis (*motA, motB, fliD, flhD*, and *flgJ*). It was reported that the velocity at which a swarming colony of *Serratia liquefaciens* colonizes a surface correlates with the level of expression of the *flhD* master operon^24^. Houry *et al*.^25^ reported that *motA* and *motB* form a bicistronic operon and encode two proteins that comprise the flagellar basal body. Therefore, the decreased motility caused by citral could be partially explained by inhibition of flagella biosynthesis or function. It was reported that trans-cinnamaldehyde significantly reduced the diameter of the motility zone of *C. sakazakii* and the expression of several genes linked to motility^17^. Burt *et al*.^26^ demonstrated that addition of carvacrol results in an aflagellate and nonmotile phenotype in *Escherichia coli* O157:H7.

A significant decrease in biofilm formation in the presence of citral at 12 °C and 25 °C was demonstarted. These two temperatures were selected by their relevance to the food industry (12 °C)^27^, and common room temperature in houses (25 °C). Moreover, citral exerted a stronger anti-biofilm ability at lower temperatures. Upadhyay *et al*.^28^ reported that trans-cinnamaldehyde, carvacrol, thymol and eugenol inhibited *Listeria monocytogenes* biofilm at 37 °C, 25 °C, and 4 °C to varying degrees. In addition, trans-cinnamaldehyde inhibited *C. sakazakii* biofilm on all matrices tested at 24 °C and 12 °C^29^. The enhanced anti-biofilm effect of citral at 12 °C could be due to the lower metabolic and growth rates of *C. sakazakii* at this temperature, which potentially result in a weaker biofilm that is more sensitive to citral^30^. Cellulose was identified as one of the exopolysaccharide (EPS) components responsible for biofilm production by members of the *Enterobacteriaceae* family, which contribute to protect cells from various environmental stressors^31^. Our results revealed that citral significantly down-regulated the expression of *bcsA* and *bcsG* that encode the cellulose biosynthesis operon^32^. We assumed that citral may influence the biosynthesis of components associated with EPS and consequently change the structure of biofilm making it more susceptible.

Citral greatly inhibited the QS-signaling system of *C. violaceum* 12472, which produced several types of *N*-acyl-_L_-homoserine lactones (AHLs), and substantially down-regulated the expression of *luxR* in *C. sakazakii* that encodes a transcriptional regulator associated with AHL type QS system^33^. As the central regulator of virulence factors, the QS system of Gram-negative bacteria produces AHLs as cell-cell signaling molecules that mediate several pathogenic processes including swarming, biofilm development and LPS release^34^. AHLs regulate bioluminescence, pigment and antibiotic production in various bacteria in response to cell density^33^. *C. sakazakii* isolates were previously reported to produce two different AHL molecules (3-oxo-C6-HSL and 3-oxo-C8-HSL)^35^. It is postulated that citral suppressed the QS by inhibiting the synthesis of the LuxR protein and interfering with bacterial cell-to-cell communication. Olivero-Verbel *et al*.^36^ reported that essential oils from *Lippia alba*, including geranial and neral, two isomeric acyclic monoterpene aldehydes of citral, had the capacity to significantly inhibit QS as observed by the reduction of violacein production in a *C. violaceum* CV026 bioassay while exhibiting insignificant effects on cell growth. Additionally, Zhang *et al*.^37^ demonstrated that citral could reduce the synthesis of AI-2, an additional QS-signaling molecule that functions in both Gram-positive and Gram-negative bacteria. Altogether, these results suggest that citral could play an important role in reducing virulence in *C. sakazakii* through QS inhibition.

Adherence to host surfaces, such as to intestinal epithelial layer, and survival in the intestinal wall with subsequent entrance into the bloodstream are both necessary for a microbe to establish infection^38^. Previous studies demonstrated that adhesion and invasion of *Salmonella enteritidis* to chicken oviduct epithelial cells were significantly reduced by carvacrol, thymol and eugenol^39^. Inamuco *et al*.^40^ reported that carvacrol did not affect adhesion but significantly reduced invasion of *Salmonella* to intestinal epithelial cells. In this study, we demonstrated that citral significantly suppressed attachment and invasion of Caco-2 cells by *C. sakazakii* and inhibited the expression of *ompA, ompX* and *uvrY*, which largely contributed to bacterial attachment and invasion of host cells^17^. Previously, outer membrane protein A (OmpA) was shown to be necessary for the colonization of *C. sakazakii* in the gastrointestinal tract and subsequent survival in blood to cause meningitis and played an important role in attachment and invasion of Caco-2 cell^41^. Additionally, Kim *et al*.^42^ observed that OmpA and OmpX contributed to the invasion of Caco-2 cells, and both are critical for the movement of *C. sakazakii* into deeper organs such as the liver and spleen. It was proposed that citral impedes the attachment and invasion of *C. sakazakii* via interference with the production of related proteins hampering bacterial invasion and survival in host cells.

Virulence studies showed that *C. sakazakii* strains were able to survive in macrophage cells, the major constituent of the innate immune system^3^. This bacterium survives and multiplies within phagocytic cells, enhancing its ability to avoid the host immune response and cause bacteremia, which could be advantageous to migrate through the blood-brain barrier endothelium^43^. Citral was found to significantly decrease the ability of *C. sakazakii* to survive and reproduce intracellularly and decreased the expression of *sod*. This gene was reported to be responsible for superoxide dismutase production, which protects bacteria from the oxidative stress generated by macrophages^44^. In a previous study, *C. sakazakii* pre-treated with trans-cinnamaldehyde was found to have difficulty surviving in human macrophages U937^17^. According to this study, citral inhibited the survival and replication of *C. sakazakii* within macrophages, diminishing its ability to withstand bactericidal activity and to evade the host immune response.

Previous studies showed that powdered infant formula is frequently contaminated with high levels of LPS, which is a major virulence factor and a key contributor to the initial adhesion of bacterial cells to a surface or host cell^45^. Our results demonstrated that citral significantly reduced the production of LPS in *C. sakazakii* and significantly down-regulated *lpxB* and *wzx*, which encode lipid A disaccharide synthase and O-antigen flippase, respectively^46^. In accordance with our findings, Amalaradjou *et al*.^17^ reported that 750 μM trans-cinnamaldehyde decreased endotoxin synthesis by 50% in *C. sakazakii*. Furthermore, Song *et al*.^47^ found that treatment of human umbilical vein endothelial cells with citral significantly inhibited TNF-α and IL-8 expression induced by LPS. The two dominant domains of LPS: lipid A and O-antigen play a major role in virulence mediating inflammatory response-induced endotoxicity and being responsible for surface attachment thus eliciting a strong antibody response from the infected host^48^. Therefore, we hypothesize that citral damages the structure of LPS and reduces its deleterious effects on the intestinal barrier integrity.

Recently, anti-virulence strategy is considered to be a promising avenue to combat bacterial infection, which was thought to impose less selective pressure on bacteria to reduce the chance of selecting resistant strains^49^. Anti-adhesion, anti-biofilm and anti-QS have all been considered promising novel therapeutics to deal with multi-resistant bacterial infections^50^. In this study, we demonstrated that SICs of citral decreased crucial virulence factors and reduced the synthesis of flagella and biofilms, and interfered with cell-to-cell signaling, all of which would contribute to diminished virulence of *C. sakazakii*. It is worth mentioning that researchers recently showed that 10-day consecutive selection with citral at SIC induces genotypic resistant *E. coli* strains^51^ and exposure to citral may enhance bacterial virulence of *Listeria spp.* in a *Caenorhabditis elegans* model^52^. Although resistant mutant was not observed in our trial experiments, further research is warranted to test this possibility to determine the appropriate application of the citral, either being developed as an alternative/supplementary strategy to control *C. sakazakii* infections or as a novel hurdle in food preservation in combination with other preservative technologies.

## Materials and Methods

### Reagents

Citral (CAS:5392-40-5) was obtained from Chengdu Must Bio-technology Co., Ltd. (Chengdu, Sichuan, China) at a HPLC purity of at least 99.2%. Citral solution were prepared in 0.1% DMSO before use. All other chemicals were of analytical grade and were unaltered.

### Bacterial Strains and Growth Conditions

*C. sakazakii* strains ATCC 29544, ATCC 29004 (ATCC, Manassas, USA) and 7–17 (our laboratory strain collection) were used in the present study. *C. sakazakii* 7–17 was originally isolated from infant formula in China and confirmed before by species-specific PCR^53^. All of the *C. sakazakii* isolates were used in MIC and SIC assays, and only ATCC 29544 was used for further experiments because it is commonly used in *C. sakazakii* virulence studies and it contains phenotypic and genotypic characteristics tested in the following experiments. The QS indicator strain *Chromobacterium violaceum* ATCC 12472 (ATCC) was used for QS inhibition assays^54^. All *C. sakazakii* strains were grown and prepared as described before^55^.

### Minimum Inhibitory Concentration and Sub-Inhibitory Concentration Determinations

The *C. sakazakii* strain was grown to an OD_600_ value of 0.1 in TSB, whereupon the same volume (125 μL) of the culture and citral solution were transferred into 96-well microtiter plates with final citral concentrations of 0 (control), 56.25 μM, 112.5 μM, 225 μM, 450 μM, 900 μM, 1800 μM, 3600 μM and 7200 μM. Bacteria was cultured at 37 °C for 24 hours, and cell growth was monitored at 600 nm as described in a previous study^56^. The MIC was defined as the lowest concentration (μM) of citral in the presence of which *C. sakazakii* failed to grow. In addition, the three highest concentrations of citral that did not inhibit bacterial growth were selected as SICs for the following assays.

### Swimming and Swarming Assay

Swimming and swarming were evaluated in LB containing different agar concentrations, as previously described^57^. For analysis of swimming ability, a medium containing 20 mL of LB broth and 0.3% (wt/vol) of agar was used. Citral was added to the warm medium (45 °C) to obtain final concentrations of 225 μM and 112.5 μM. Subsequently, the plates were allowed to dry for 1 hour at 25 °C before use. Five microliters of each *C. sakazakii* culture (~6.0 log CFU) were inoculated at the center of this semisolid medium and the plates were incubated at 37 °C for 7 hours. After this, the diameter of the bacterial halo was recorded. The medium without citral was used as a control.

For analysis of bacterial swarming ability, a medium containing 20 mL of LB broth, 0.5% (wt/vol) agar and 0.5% (wt/vol) glucose was used. Five microliters of *C. sakazakii* culture (~6.0 log CFU) were stabbed into the semisolid medium without and with citral at 225 μM and 112.5 μM. The plates were incubated upside down at 37 °C for 20 hours. The size of the swarm area in the presence or absence of citral was calculated using AutoCAD.

### Visualization of Flagella by Electron Microscopy

Transmission electron microscopy was used to examine the morphology of flagella. *C. sakazakii* strain ATCC 29544 cells were taken from swimming plates at different citral concentrations (0, 112.5 μM and 225 μM) and suspended in sterile PBS. One drop of culture was placed on a Formvar-coated grid (Electron Microscopy Sciences) for 20 min. Excess liquid was wiped off and the grid was stained with 1% sodium phosphotungstic acid (pH 6.8) for 1 min and washed three times with distilled water. All specimens were examined with a transmission electron microscope (HT7700, Hitachi, Tokyo, Japan).

### Specific Biofilm Formation (SBF) Inhibition Assay

Biofilm formation assays were performed according to the method described by Naves, *et al*.^58^ with minor modifications. Overnight cultures of *C. sakazakii* strains ATCC 29544 were centrifuged (4 °C, 5000 × g, 10 min) and re-suspended in TSB. Then, 250 μL of the cell suspension (OD_600_ = 1) were inoculated in sterile 96-well microtiter plates, and citral was added to each of the wells to obtain a final concentration of 0, 112.5 μM and 225 μM. Non-inoculated TSB was used as a control. Six samples were included for each treatment. The plates were incubated at 25 °C or 12 °C for 24, 48 and 72 hours without agitation. At each time points, the optical densities (ODs) of cell growth were measured at 630 nm using a microplate spectrophotometer (Model 680; Bio-Rad, Hercules, CA, U.S.A.). The suspension was then removed and the wells were rinsed once with 350 μL of distilled water. After being air dried for 30 minutes, the wells were stained with 250 μL of 1% crystal violet (wt/vol) (Tianjin Kermel Chemical Regent Co., Ltd, Tianjin, China) for 20 minutes at room temperature. To remove the non-conjugated colorant, the wells were rinsed three times with 350 μL of distilled water. After being air-dried for 30 minutes, the adhered colorant was solubilized in 250 μL of 33% (vol/vol) glacial acetic acid and incubated for 20 minutes at room temperature. Then the OD of each well was measured at 570 nm. The SBF was calculated by correcting the OD_570_ with the OD_630_.

### Quantitative QS Inhibition Assay

The effect of citral on the QS inhibitory activity was measured by quantifying the violacein production with the indicator strain *C. violaceum* ATCC 12472^59^. First, the inhibitory effect of citral on the growth of *C. violaceum* was studied to determine the SIC to be used in further experiments. The two highest concentrations of citral that did not inhibit *C. violaceum* growth after 24 hours of incubation were selected as SICs for this study. Duplicate wells were included for each citral concentration and the experiment was repeated three times.

A flask incubation assay was used to quantify the QS-inhibitory activity of citral. An overnight culture of *C. violaceum* was diluted to an OD_600_ of 0.5. Volumes (30 mL) of LB broth that contained different concentrations of citral were placed into separate flasks. Each flask was inoculated with 100 μL of culture. The flasks were incubated at 30 °C for 24 hours with 150 rpm. The violacein extraction and quantitation were carried out as previously described by Choo *et al*.^60^ with minor modifications. Briefly, 5 mL of the *C. violaceum* culture was centrifuged (5000 × g, 5 min, 4 °C) to precipitate insoluble violacein and the culture supernatant was discarded. Then, 1 mL of DMSO was added to the pellet and the solution was vortexed vigorously for 1 min to completely solubilize violacein. The solution was then centrifuged at 5000 × g for 10 min to remove the cells. Two hundred microlitres of the violacein-containing supernatants were added to 96-well microtiter plates and the absorbance of violacein-containing supernatants was read with a microplate spectrophotometer (Model 680; Bio-Rad, Hercules, CA, U.S.A.) at a wavelength of 585 nm.

### Cell Culture

Human colon adenocarcinoma cell line Caco-2 was maintained in Dulbecco’s Modified Eagle Medium (DMEM) (Gibco, Grand Island, NY, U.S.A.). Murine macrophage cell line RAW 264.7 cells were grown in RPMI 1640 (Gibco). Both media were supplemented with 10% (vol/vol) fetal bovine serum (FBS) (Hyclone, Logan, UT, U.S.A.), 1% (vol/vol) nonessential amino acids (Gibco) and 1% (vol/vol) double antibiotic solution (100 U/mL penicillin and 100 μg/mL streptomycin; Hyclone). Maintenance of the cell lines and subsequent experiments were carried out at 37 °C in a humidified atmosphere containing 5% CO_2_.

### Adhesion and Invasion Assay

The effect of citral on the adhesion and invasion of *C. sakazakii* was investigated by using Caco-2 cells, as described in a previous study^17^. Trypsin-treated cells were seeded in 24-well tissue culture plates (Nunc) containing supplemented DMEM (10^5^ cells per well) and incubated for 24 hours. *C. sakazakii* was grown to its mid-log phase with and without SICs of citral (56.25 μM, 112.5 μM and 225 μM), then centrifuged and re-suspended in cell culture media without antibiotics. Then, the Caco-2 cells were rinsed with PBS and inoculated in the medium with 10^6^ CFU (10 MOI) of the *C. sakazakii* suspension. The tissue culture trays were centrifuged at 600 × g for 5 minutes and incubated at 37 °C in a humidified, 5% CO_2_ incubator.

For the adhesion assay, the infected cell monolayers were rinsed three times in PBS after 1 hour of incubation, and lysed with 0.1% Triton X-100 (Amresco, Solon, OH, U.S.A.). The number of viable adherent *C. sakazakii* was determined by the serial dilution and plating on TSA agar plates and incubated at 37 °C for 24 hours before counting. For the invasion assay, the cell monolayers were incubated for 1 hour following infection, rinsed three times in PBS, and incubated for another 30 minutes in whole media containing gentamicin (100 μg/mL; Amresco) to kill the extracellular bacteria. Finally, the wells were washed with PBS three times. The numbers of internalized *C. sakazakii* were determined as described in the adhesion assay. The numbers of bacteria in the treatments were expressed as a percentage relative to that of the control medium.

### Effect of Citral on Intracellular Survival and Replication of *C. sakazakii* in RAW 264.7 Cells

The murine macrophage cell line RAW 264.7 cells were maintained in RPMI 1640 medium with 10% FBS. Twenty-four hours prior to infection, activated cells were seeded in 24-well tissue plates (10^5^ cells per well) and cultured at 37 °C under 5% CO_2_. *C. sakazakii* was grown to its mid-log phase at various concentrations of citral (0, 56.25 μM, 112.5 μM and 225 μM). Then the RAW 264.7 cells were washed gently with PBS and infected with 10^6^ CFU (MOI = 10) of *C. sakazakii*. The plates were incubated for 45 minutes at 37 °C with 5% CO_2_. After incubation, RAW 264.7 cells were re-suspended in RPMI 1640 containing 1% FBS with gentamicin (100 μg/mL) and incubated at 37 °C with 5% CO_2_ for 30 minutes.

For intracellular survival assays, the cells were then washed three times with PBS and lysed with 0.1% Triton. After dilution (PBS 1:10), the samples were enumerated on TSA plates. The results were presented as the number of intracellular *C. sakazakii* cells after citral treatment. For replication assays, each well containing bacterial cells was replenished with RPMI 1640 containing 1% FBS with gentamicin (10 μg/mL) and incubated at 37 °C with 5% CO_2_ for either 24 or 48 hours. Cell washing, lysis and plating procedures were identical to those used in the analysis of bacterial survival. All assays were conducted in triplicate and repeated at least three times on different days.

### *C. sakazakii* Endotoxin Assay

ToxinSensor Chromogenic LAL Endotoxin Assay Kit (GenScript, Piscataway, NJ, U.S.A.) was used according to the method of Amalaradjou, *et al*.^17^. Overnight cultures of *C. sakazakii* strains ATCC 29544 was centrifuged (4 °C, 5000 × g, 10 min) and re-suspended in TSB. Then, 50 μL of the cell suspension (OD_600_ = 0.5) and citral solution was added in 30 mL TSB to obtain a final concentration of 0, 112.5 μM and 225 μM at 37 °C to its mid-log phase. Non-inoculated TSB was used as a control. Samples were treated following manufacturer instructions and analyzed by a microplate spectrophotometer (Model 680; Bio-Rad, Hercules, CA, U.S.A.).

### Quantification of *C. sakazakii* Virulence Gene Expression

The *C. sakazakii* strain was grown without and with SICs of citral (112.5 μM and 225 μM) in TSB at 37 °C to its mid-log phase. Next, the bacteria were centrifuged (5000 × g, 5 min, 4 °C) and re-suspended in PBS. The total RNA was extracted with the RNAprep Pure Bacteria Kit (Tiangen, Beijing, China) according to the manufacturer’s protocol. RNA concentrations were measured with a nucleic acid and protein spectrophotometer (Nano-200; Aosheng Instrument Co., Ltd., Hangzhou, China). First-strand cDNA was synthesized from 3 μL of each RNA sample in a 10 μL reaction volume using the PrimeScript RT Reagent Kit (Takara, Kyoto, Japan) according to manufacturer directions. The primer sequences used for RT-PCR are listed in Table 2. RT-PCR was performed in a 25-μL system using SYBR Premix Ex Taq II (Takara). The cycling conditions included 1 cycle of 95 °C for 30 seconds, 40 cycles of 95 °C for 5 seconds and 60 °C for 30 seconds, and dissociation steps of 95 °C for 15 seconds and 60 °C for 30 seconds. All samples were analyzed in triplicate and normalized to the endogenous control (ESA_04030) gene. Samples were run on the IQ5 system (Bio-Rad Laboratories, Hercules, CA, U.S.A.) and the expression of target genes versus ESA_04030 gene were determined as previously described^57^.

### Statistical Analysis

All experiments were carried out in independent triplicate. Data were expressed by mean ± standard deviation and analyzed with IBM SPSS v.19.0 software (version 19.0; SPSS Inc, IBM Co., Armonk, NY,USA). Differences were considered to be significant when *P* ≤ 0.05.

## Additional Information

**How to cite this article**: Shi, C. *et al*. Inhibition of *Cronobacter sakazakii* Virulence Factors by Citral. *Sci. Rep.* **7**, 43243; doi: 10.1038/srep43243 (2017).

**Publisher's note:** Springer Nature remains neutral with regard to jurisdictional claims in published maps and institutional affiliations.

## Figures and Tables

**Table 1:** Inhibition of *C. sakazakii* ATCC 29544 biofilm formation by sub-inhibitory concentrations of citral at 25 °C and 12 °C.

SBF	25 °C			12 °C		
	CK	112.5 μM	225 μM	CK	112.5 μM	225 μM
Time (h)						
24	1.49 ± 0.11	0.63 ± 0.07**	0.49 ± 0.07**	1.18 ± 0.15	0.80 ± 0.10	0.70 ± 0.22*
48	1.31 ± 0.27	0.87 ± 0.15*	0.40 ± 0.22**	3.32 ± 0.32	2.29 ± 0.38*	1.83 ± 0.21*
72	0.67 ± 0.02	0.39 ± 0.02**	0.20 ± 0.03**	1.50 ± 0.05	0.98 ± 0.17**	0.55 ± 0.04**

Values are shown as mean ± the standard deviation of three independent experiments. ^*^P ≤ 0.05, ^**^P ≤ 0.01 compared to *C. sakazakii* cells without citral treatment (CK).

**Table 2:** Fold change in the expression level of *C. sakazakii* ATCC 29544 virulence-associated genes in the presence and absence of citral.

Target gene	Sequence of primers (5′-3′)	Relative gene expression at		Reference
		112.5 μM	225 μM	
ESA_04030	F, CCAGGGCTACACACGTGCTA	1	1	29
	R, TCTCGCGAGGTCGCTTCT			
*bcsA*	F, CACGATGGTGGCGTTGTTC	−2.65 ± 0.27^b^	−3.04 ± 0.59^b^	29
	R, CCTTTGGCGGGTGACGTTAA			
*bcsG*	F, ACGACTGGCTAACAGCTTTTAC	−1.72 ± 0.16^b^	−2.29 ± 0.70^b^	29
	R, GCCGGGAAGGTTGTCTGA			
*flhD*	F, CGATGTTTCGCCTGGGAAT	−2.66 ± 0.63^b^	−2.74 ± 0.27^b^	29
	R, AGAGTCAGGTCGCCCAGTGT			
*fliD*	F, AAAACCGCAACATGGAATTCA	−1.05 ± 0.05	−1.24 ± 0.17^b^	29
	R, CCGCAAACGCGGTATTG			
*flgJ*	F, GACGGCGGGCAAAGG	−1.13 ± 0.19	−7.66 ± 1.65^b^	29
	R, GCCGCCCATCTGTTTGAC			
*motA*	F, GGTGTGGGTGCGTTTATCGT	−1.23 ± 0.20	−1.30 ± 0.11^b^	29
	R, GCCTTCAGCGTGCCTTTG			
*motB*	F, ACGGCTCGTGGAAAATCG	−1.15 ± 0.06^a^	−1.47 ± 0.14^b^	29
	R, CCAGGAAGAAGGCCATCATG			
*luxR*	F, TGTGCGTTCGCCATCCT	−2.03 ± 0.10^b^	−2.28 ± 0.09^b^	29
	R, TGGTGTGCAGCGTCAGTTTT			
*lpxB*	F, GCACGACACTTTCCGTAAACTG	−4.18 ± 0.28^b^	−5.70 ± 0.43^b^	17
	R, CGCCTGTTCATCGGCATT			
*ompA*	F, GGCCGCATGCCGTATAAA	−1.36 ± 0.07^b^	−3.53 ± 0.06^b^	17
	R, GCTGTACGCCCTGAGCTTTG			
*ompX*	F, GTCTTTCAGCACTGGCTTGTGT	−1.08 ± 0.25	−1.81 ± 0.07^b^	17
	R, GGTGCCAGCAACAGCAGAA			
*sod*	F, CGAATCTGCCGGTTGAAGA	−1.54 ± 0.06^b^	−2.22 ± 0.52^b^	17
	R, CTTGTCCGCCGGAACCT			
*uvrY*	F, GCGAGGACGCCATCAAAT	−1.27 ± 0.09^b^	−3.47 ± 0.72^b^	17
	R, ATCCATCAGCACCACATCCA			
*wzx*	F, TGCTTGGGCAGGTACAAAGTG	−2.03 ± 0.07^b^	−4.51 ± 0.39^b^	17
	R, CCCTACGGGTGCAGTCACA			
*galE*	F, CTTGAGTATTACGACAACAACG	−4.42 ± 0.67^b^	−10.55 ± 0.86^b^	This study
	R, GAAACTTTCGACATAAGGGAT			
*kpsT*	F, ATTGGCGGGACGGATAA	−1.16 ± 0.08^b^	−11.51 ± 1.21^b^	This study
	R, TCGTCCACCAGGTAGTAGTCA			
*iucC*	F, CGATGAGACCTGGACATTTGA	−1.37 ± 0.07^b^	−6.50 ± 0.46^b^	This study
	R, ACGCCCTTTGTTGAAGATAA			
*eitA*	F, CGCTGACCCTTGATAACTG	−1.64 ± 0.11^b^	−1.99 ± 0.18^b^	This study
	R, TTCGTAACGCAGACACCAG			

^a^*P* ≤ 0.05, ^*b*^*P* ≤ 0.01. ^*c*^F, forward; R, reverse.
